# Optimizing the Definitions of Stroke, Transient Ischemic Attack, and Infarction for Research and Application in Clinical Practice

**DOI:** 10.3389/fneur.2017.00537

**Published:** 2017-10-18

**Authors:** Anne L. Abbott, Mauro Silvestrini, Raffi Topakian, Jonathan Golledge, Alejandro M. Brunser, Gert J. de Borst, Robert E. Harbaugh, Fergus N. Doubal, Tatjana Rundek, Ankur Thapar, Alun H. Davies, Anthony Kam, Joanna M. Wardlaw

**Affiliations:** ^1^Department of Epidemiology and Preventive Medicine, School of Public Health and Preventive Medicine, Monash University, Melbourne, VIC, Australia; ^2^The Neurology Department, The Alfred Hospital, Melbourne, VIC, Australia; ^3^Neurological Clinic, Marche Polytechnic University, Ancona, Italy; ^4^Department of Neurology, Academic Teaching Hospital Wels-Grieskirchen, Wels, Austria; ^5^Queensland Research Centre for Peripheral Vascular Disease, College of Medicine and Dentistry, James Cook University, Townsville, QLD, Australia; ^6^Department of Vascular and Endovascular Surgery, The Townsville Hospital, Townsville, QLD, Australia; ^7^Cerebrovascular Program, Neurology Service, Department of Medicine, Clínica Alemana de Santiago, Facultad de Medicina Clínica Alemana – Universidad del Desarrollo, Santiago, Chile; ^8^Department of Vascular Surgery, University Medical Centre of Utrecht, Utrecht, Netherlands; ^9^Department of Neurosurgery, Penn State University, State College, PA, United States; ^10^Centre for Clinical Brain Sciences, University of Edinburgh, Edinburgh, United Kingdom; ^11^Department of Medicine, Elderly Royal Infirmary of Edinburgh, Edinburgh, United Kingdom; ^12^Department of Neurology, Miller School of Medicine, Miami, FL, United States; ^13^Imperial College Healthcare NHS Trust, London, United Kingdom; ^14^Imperial College, London, United Kingdom; ^15^Academic Section of Vascular Surgery, Department of Surgery and Cancer, Imperial College School of Medicine, Charing Cross Hospital, London, United Kingdom; ^16^Department of Radiology, Alfred Health, Melbourne, VIC, Australia; ^17^Division of Neuroimaging Sciences, Centre for Clinical Brain Sciences, UK Dementia Research Institute at the University of Edinburgh, Edinburgh, United Kingdom

**Keywords:** stroke, transient ischaemic attack, infarction, asymptomatic carotid stenosis, public health practice

## Abstract

**Background and purpose:**

Until now, stroke and transient ischemic attack (TIA) have been clinically based terms which describe the presence and duration of characteristic neurological deficits attributable to intrinsic disorders of particular arteries supplying the brain, retina, or (sometimes) the spinal cord. Further, infarction has been pathologically defined as death of neural tissue due to reduced blood supply. Recently, it has been proposed we shift to definitions of stroke and TIA determined by neuroimaging results alone and that neuroimaging findings be equated with infarction.

**Methods:**

We examined the scientific validity and clinical implications of these proposals using the existing published literature and our own experience in research and clinical practice.

**Results:**

We found that the proposals to change to imaging-dominant definitions, as published, are ambiguous and inconsistent. Therefore, they cannot provide the standardization required in research or its application in clinical practice. Further, we found that the proposals are scientifically incorrect because neuroimaging findings do not always correlate with the clinical status or the presence of infarction. In addition, we found that attempts to use the proposals are disrupting research, are otherwise clinically unhelpful and do not solve the problems they were proposed to solve.

**Conclusion:**

We advise that the proposals must not be accepted. In particular, we explain why the clinical focus of the definitions of stroke and TIA should be retained with continued sub-classification of these syndromes depending neuroimaging results (with or without other information) and that infarction should remain a pathological term. We outline ways the established clinically based definitions of stroke and TIA, and use of them, may be improved to encourage better patient outcomes in the modern era.

## Introduction

It is vital that everyone, including patients, carers, clinicians, researchers, policy makers, health-care funders, and the general public, know what is meant by “stroke” because everyone has a role to play in optimizing action to reduce its impact. Stroke imposes huge socioeconomic burdens, being persistently among the top three causes of disability and premature mortality world-wide (1, 2). Further, stroke is highly preventable and effective hyperacute therapies are now available (3–6). As explained in this report, stroke has been a clinically based diagnosis, describing the nature and duration of deficits, with sub-categorization according to neuroimaging results. This remains appropriate (despite advances in neuroimaging and therapies) because the clinical status matters most to patients. Further, the clinical status (or anticipation of it) is the reason for, and target of, any treatment. The clinical status must be weighed up against the risks of treatment and has been the primary outcome measure in studies of prognosis and therapy. As explained below, there have been proposals to change the definitions of stroke, transient ischemic attack (TIA), and infarction to ones dominated by neuroimaging results. In this report, we present these proposals and the reasons given for them. Further, we use our own experience in clinical practice and research to demonstrate how these proposals are problematic. Finally, we hypothesize regarding how the existing clinically based definitions of stroke and TIA may be optimized in the modern era.

## The Meaning of Stroke, TIA, and Infarction

### The Established Clinically Based Definitions

For several decades, the meaning of “stroke” (in scientific and lay literature) has most often been consistent with the 1980 World Health Organization (WHO) definition as “rapidly developed clinical signs of focal (or global) disturbance of cerebral function, lasting more than 24 h or leading to death, with no apparent cause other than of vascular origin” (7). This definition describes a clinical syndrome. It includes no details regarding vascular mechanism because its utility lies in optimizing the identification of all deficits caused by cerebral ischemia (reduced blood supply) or hemorrhage. Both of these fundamental conditions produce similar clinical features and require urgent medical attention. The stroke mechanism is determined, as clearly as possible, once such attention is provided. Of note, “global” disturbance of cerebral function referred only to patients with subarachnoid hemorrhage and without “focal” neurological deficits (7) and is often omitted in practice. However, although features suggesting global cerebral disturbance (such as impaired or loss of consciousness) are not commonly associated with focal, intrinsic cerebrovascular ischemia, they may occur (8–10), just as they can with subarachnoid hemorrhage (11).

Stroke has been distinguished from TIA which most often has been interpreted as the 1975 National Institutes of Health (NIH) definition as “episodes of temporary and focal cerebral (including retinal) dysfunction of vascular origin, rapid in onset which commonly last 2–15 min but occasionally up to a day (24 h)” where “resolution is swift and leaves no permanent [clinical] neurologic deficit” (12). This definition was formulated recognizing that duration of clinical features associated with untreated cerebrovascular compromise follows a spectrum. Most deficits which resolve spontaneously within 24 h, do so within the first minutes or hours (13–15). Using a standard time-point to differentiate short from long-term deficits provides a “common” basis for “collecting” information about the large population with clinical deficits that resolve quickly with no detectable residuum, whether or not hyperacute treatment is used (12, 16, 17). As discussed later, strengths of 24-h time-point include that it is relatively easily measured, it has been the main standard to date and it can be used as an important outcome measure of hyperacute treatment or prevention strategies.

The WHO and NIH definitions of stroke and TIA are concise and dependent only on recognition of fundamental clinical features. Therefore, they are easily accessible to the public and professionals anywhere in the world following some relatively basic education. They are completely amendable to sub-classification using additional information, including neuroimaging results. They facilitate an orderly framework for proceeding from clinical stage to most likely mechanism and best management (12). As discussed later, improvement can be made by providing detail about typical clinical presentations and specifying the inclusion, or not, of stroke and TIA of the retina and/or spinal cord or syndromes due to arterial and/or venous infarction. However, these core definitions have predominated in the past decades of research and communication of research findings. Consequently, they hold the strongest overall value for the identification, management, prognostication, and prevention of acute cerebrovascular emergencies.

### Proposals to Change to Imaging-Dominant Definitions

Changes to these established definitions have been proposed by representatives of the American Heart and Stroke Associations (AHA/ASA) and others in three publications (18–20). Although suggestions made differ, there is a common proposal to shift from clinically based definitions of stroke and TIA (describing the presence and duration of characteristic neurological deficits) to definitions according to neuroimaging alone. A further proposal is that imaging findings be equated with infarction. However, to date “infarction” has been a pathological term used to describe tissue that has lost its blood supply for long enough to undergo ischemic necrosis (death) with characteristic macroscopic and microscopic findings (21). Infarction can only be accurately identified using histopathologic techniques.

As explained in below, we have observed that attempts to use the AHA/ASA proposed new definitions are causing confusion and chaos. This is not surprising given that the proposed new “definitions” are ambiguous, scientifically incorrect, research disrupting, otherwise clinically unhelpful and do not solve the problems they were proposed to solve. The value of brain imaging in the management of patients with clinical features of acute, focal central-neurovascular compromise is not being questioned here. It is the proposal to change from clinically based to imaging-dominant definitions that is problematic.

## The Proposals are Ambiguous

### 2002 Proposals

The proposed new definitions of stroke and TIA fail because of ambiguity (18–20). For example, it was proposed in 2002 that TIA now be defined as “a brief [not defined] episode of neurologic dysfunction caused by focal brain or retinal ischemia with clinical symptoms typically lasting less than one hour and without evidence of acute infarction [not defined]. The corollary is that persistent [not defined] clinical signs or characteristic imaging abnormalities [not defined] define infarction- that is stroke.” (18) This text contains no valid definitions because the duration of symptoms and what constitutes infarction (or, more appropriately, imaging evidence of infarction) were not specified.

### 2009 Proposals

The 2009 suggestion by Easton et al. added to the ambiguity by removing more detail: “TIA: a transient [not defined] episode of neurological dysfunction caused by focal brain, spinal cord or retinal ischemia without acute infarction [not defined].” (19) Further, it was proposed that ischemic stroke be defined as “infarction [not defined] of the central nervous system” whereas TIA be defined as “symptomatic ischemia [not defined] without infarction [not defined].” Equivocacy was amplified by inconsistency. Initially it was proposed that that ischemic stroke *requires* the presence of “infarction” and later, that some ischemic strokes will be rendered “only on the basis of clinical features.” It was also proposed, perhaps clarifying the 2002 proposal, that “ischemic strokes” refer to “symptomatic or silent” brain imaging abnormalities (19). The place of primary cerebral hemorrhage in the definition of stroke was not addressed in either publication.

### 2013 Proposals

In the third publication (2013) the suggested new definitions differed again with shifts back to a clinical basis and acknowledgment of the pathological dimension of cerebrovascular disease (20). However, as in 2002 and 2009, the proposed new 2013 definition of infarction remains unspecified: “infarction is brain, spinal cord or retinal cell death attributable to ischemia based on patholological [not defined], imaging [not defined] or other objective evidence [not defined] and/or clinical evidence [not defined].” There again was inconsistency with a later proposal that central nervous system infarction be defined using an explicit “vascular distribution” and a “≥24 h” duration for “clinical evidence.” Further, a 405 word stroke definition was proposed, too long for everyday use. These AHA/ASA proposals cannot provide standardization, only confusion. This is reason enough that these proposals should not be accepted.

## The Proposals are Scientifically Incorrect

The fundamental AHA/ASA proposal is that brain imaging must be used, or may be used alone, to diagnose stroke (the patient’s clinical deficits) and infarction (ischemic death of central nervous system tissue). Both aspects are scientifically incorrect. Brain imaging findings do not always correlate with clinical deficits or infarction. As mentioned, the timing and nature of brain imaging proposed to “identify” stroke, TIA, and infarction were not specified (18–20). However, early echo-planar diffusion-weighted (DWI) magnetic resonance imaging (MRI) is especially implicated because of its superiority in detecting “positive” evidence of ischemia (rather than simply excluding alternative pathologies) compared to other MRI sequences (22–24) and, in particular, compared to computed tomography (CT).

### Limited Sensitivity of Cerebral Neuroimaging

Reported sensitivities of non-contrast CT brain performed 0–6 h after clinical onset in detecting “positive” evidence of brain ischemia (such as hypo-attenuation, loss of white/gray matter differentiation, focal cortical swelling, hyper-dense arterial signs and/or compression of adjacent structures) vary from 12 to 66% (25–27). This can be improved somewhat by using contrast and CT perfusion (CTP) imaging (28). However, CTP is limited in detecting change indicative of cerebral ischemia, including infarction, and is susceptible to larger measurement errors than DWI (29, 30). “Positive” DWI evidence of acute brain ischemia *may* sometimes be seen minutes after arterial compromise as hyperintensity (31, 32). From animal models, early DWI hyperintensity is consistent with increased restriction of extra cellular water diffusion as water enters cells due to impaired membrane homeostasis (cell swelling or cytotoxic edema) (31, 33–35). However, as well recognized, all brain imaging (including DWI) is limited in characterizing ischemic tissue and stroke syndromes (20, 29, 36). Correlation of clinical features, imaging, and biological sampling (including, on occasion, brain tissue) is essential for maximizing diagnostic accuracy because all three dimensions are limited individually and provide additive value (37–42).

For example, reported DWI *sensitivities* (with 1.5–3 T field strength) for showing “positive” evidence of corresponding brain ischemia in patients imaged 0–6 h after clinical onset vary from 73 to 100% (25, 27, 42–44) and from 0 to 100% if imaged 0–90 or >14 days after clinical onset (including personal communication from Alejandro Brunser) (22, 23, 26, 27, 39, 41, 45–51). In some of these studies, imperfect sensitivity (0–100%) related specifically to patients with ischemic stroke defined by typical clinical deficits lasting >24 h with/without corresponding lesions on repeat DWI, T2, or fluid attenuation inversion recovery (FLAIR) MRI performed >24 h after clinical onset (23, 39, 45, 46, 48, 51). Further, the smallest infarcts are sometimes below the structural resolution (approximately 1 mm^3^) of 1.5–3.0 T MRI and although the resolution of 7 T MRI is better, it is not perfect (36, 52–54).

Diffusion-weighted negativity is more likely with brief deficits (48, 55), imaging performed within 3–72 h (27, 46, 50) or after 2–3 weeks of clinical onset (39, 45), with acute on chronic infarction (47), less severe clinical features (for instance, NIHSS score < 4) (27, 39, 42), small areas of imaging abnormality (<5–10 mm) (46, 50), female gender (39), younger age (39), location in the posterior circulation (23, 27, 42, 46, 47, 49, 50), strokes attributable to small vessel disease (42, 47, 49), and less experienced image interpretors (25). DWI negativity is also more likely with increasing number of such predictors (27). Sensitivity may be improved by additional MR perfusion imaging (34, 42, 56). However, as commonly implemented, this requires gadolinium-based contrast and is limited by other factors, including unreliable result interpretation (29, 57). Stroke patients with corresponding positive DWI evidence of brain ischemia are more likely to have recurrent DWI “lesions” than stroke patients without corresponding positive DWI evidence of brain ischemia (51). This may indicate an individual susceptibility to developing DWI abnormalities (51).

If DWI is negative, other MRI sequences (such as T2 or FLAIR) may show signs of corresponding brain ischemia (including focal hyperintensity), particularly when patients are imaged sub-acutely (up to 8 weeks after clinical onset) (37, 39, 40, 46). Conversely, with subacute presentations, DWI may be “positive” when T2 is negative, especially with smaller lesions (24). However, MRI (using DWI and/or T2 and/or FLAIR and/or T1) performed 0–27 days from clinical onset has shown no evidence of corresponding ischemia in 3–29% of patients with typical stroke deficits lasting >24 h (23, 39, 46, 51).

### Limited Specificity of Cerebral Neuroimaging

In addition, MRI-DWI has imperfect *specificity* for identifying areas of acute cerebral ischemia. Non-stroke conditions, including hypoglycemia, viral encephalitis, abscesses, cysts, old infarcts, multiple sclerosis, trauma, migraine, transient global amnesia, mitochondrial disorders, prion disease, methanol poisoning, cellular tumors (such as lymphoma), seizures, and other conditions may produce DWI hyperintensity (38, 41, 47, 51, 58–69). While the distribution of hyperintensity usually differs somewhat with different pathologies, overlap occurs (38, 66). For, example hypoglycemia can exactly mimic focal ischemia on all sequences, including DWI (66). Reported DWI specificities in detecting evidence of corresponding brain ischemia in patients imaged 0–12 h from clinical onset vary from 75 to 95% (27, 42, 47) and from 96 to 100% in patients imaged 0–8 days from clinical onset (25, 27, 41, 47).

Further, DWI is not reliable for estimating “lesion” age. DWI can be “positive” for up to several weeks or sometimes many months after acute ischemic stroke symptom onset before normalization, with notable lack of predictability (24, 37, 70, 71). DWI lesions may be hyper-intense in the subacute phase due to increased T2 signal (T2 shine through). Apparent diffusion coefficient (ADC) values can help interpret DWI because they do not have a T2 component and are typically reduced, producing hypodense images, in the first 7–10 days of clinical onset. They then typically become iso-intense (pseudo-normalization) and then hyper-intense compared to normal brain (22, 70, 72). However, ADC values may take hours (73) or many months (37) to normalize with notable inter-individual variation. No ADC values (even if severely reduced) reliably define infarction, even though trends have been observed (29, 35, 74, 75). Further, ADC values can be reduced in many conditions other than acute brain ischemia (67).

There is other evidence that MRI (including DWI) does not just detect infarction. It may also detect “penumbra” (salvageable tissue) as evidenced by patients with acute deficits and corresponding DWI changes who make a full spontaneous clinical recovery, within minutes to days of onset (56, 74, 76–78). In addition, DWI abnormalities can completely vanish and leave no signal abnormalities on repeat DWI, T1, T2, and/or FLAIR imaging several days, weeks or months later, whether or not deficits resolve spontaneously (23, 74, 76, 79–81). Further, penumbra or infarcted tissue may be missed by initial MRI because “infarct” growth (as defined using MRI) may occur in about 50% of patients without diffusion/perfusion imaging mismatch (29, 82). In addition, DWI lesions may first appear only days after isolated perfusion deficits which are seen within 24 h of the clinical onset of suspected stroke or TIA (83). These populations require further study.

Studies of DWI reversal after thrombolysis have usually focused on the first 1–7 days after clinical onset, demonstrating some or complete “early sustained” DWI reversal over sequential scans in 0–50% of patients or “DWI lesions” (79, 84–87). To account for possible confounding effects of DWI “pseudo-normalization” in the first 7–10 days, partial late and sustained DWI reversal has also been described involving an average of 20% of the initial DWI hyperintensity volume in 7% of patients 30 or 90 days after thrombolysis (44). DWI reversal after thrombolysis (by varying definitions) is more likely, or more noticeable, with absence of baseline perfusion deficits, thrombolysis <3 h, evidence of reperfusion or small lesions (44, 85, 88, 89). In some studies, DWI reversal within a week of clinical onset was associated with better clinical outcomes (79, 86–90).

### Limited Sensitivity and Specificity of Spinal Cord Neuroimaging

Research regarding spinal cord stroke or infarction and imaging is less common than for cerebrovascular disease and challenged by perceived rarity of spinal cord stroke, variable clinical manifestations, complex spinal arterial supply, technical challenges, and overall limitations in differentiating ischemia from other pathological processes (91, 92). Sensitivities of 67–100% have been reported for initial plus/minus repeat T2 MRI in detecting evidence of ischemic injury in patients with sudden spinal cord deficits of no other apparent cause (91, 93, 94). As with cerebrovascular disease, negative T2 is more likely with less severe deficits or hyperacute imaging (93, 94). Spinal DWI is an emerging application which appears to improve early diagnostic sensitivity (92). Pathologically diagnosed lacunar type cord infarcts are common in patients dying of cerebrovascular disease, indicating scope for improved case ascertainment (95).

### The Important Role of the Neuropathologist

Finally, the 2002 and 2009 AHA/ASA proposals, in particular, abolish the role of the pathologist in stroke medicine. Brain tissue sampling carries risk and is not usually required to optimize outcomes (38). However, occasionally a biopsy is the only mechanism for a correct diagnosis when the cause of a patient’s neurological deficit is unclear or atypical from the clinical and imaging information (38). Maintaining the pathological definition of infarction, the clinical definition of stroke and TIA and acknowledging the additive information from imaging recognizes that imaging does not always accurately identify ischemia or infarction and that not all ischemic events are symptomatic or produce specific clinical or imaging features (36, 52, 54, 96, 97).

Brain images (including DWI, ADC and perfusion measurements) in patients with stroke symptoms are only snap shots of a complex dynamic process. Although helpful with other information, they alone are not fail-safe diagnostic markers of clinical status, infarction, “lesion core” or tissue at risk (29, 34, 57, 73, 75, 98). The AHA/ASA proposals to use imaging to replace clinical and pathological information are scientifically incorrect and confuse existing, purposeful terminology.

## The Proposals are Research Disrupting

### Problems Making Measurements and Comparisons

Research is about discovering new knowledge which is applied in clinical practice to improve patient outcomes. New knowledge discovery and its application require standardization of terminology and concepts, which is removed by the AHA/ASA proposed definitions. Further, over-reliance on a test (such as DWI) which is highly variable with respect to availability, acquisition methods and interpretation is likely to reduce diagnostic accuracy (99). Stroke and TIA incidence and prevalence measurements will artificially change in proportion to the use of MRI and attempted use of the proposed definitions (99–101) and most likely in ways that are difficult or impossible to decipher depending on when and how MRI is used and to the extent research methods are described. The proposed shift to an imaging-dominant diagnostic approach will also artificially change outcome measurements. For instance, classifying clinical deficits lasting less than 24 h and accompanied by a DWI lesion as stroke would increase the proportion of patients with good outcomes, according to the availability of neuroimaging and not through any health-care change. Countries with good access to MRI would likely see “improved” stroke outcomes compared to those without.

### Particular Problems with Low Rates and Differentiating Transient Events with an Example Regarding Stroke Risk Associated With Asymptomatic Carotid Stenosis

The AHA/ASA focus on imaging is causing confusion in differentiating rates of extremely transient neurological deficits compared to long-term and permanent deficits caused by ischemia. This is particularly problematic in situations where event rates are low and the principal objective is to prevent long-term neurological deficits, as with carotid procedures. For example, an attempt to use an AHA/ASA imaging-dominant “definitions” caused confusion and disruption while updating a 2009 meta-analysis (3) of temporal change in the average annual ipsilateral stroke rate in patients with 50–99% asymptomatic carotid stenosis given medical (non-procedural) treatment alone (102). The original 2009 methods were repeated with inclusion of studies published from January 2009 to December 2013 and identified using all PubMed listings with “carotid” in the title (8,480 listed after duplicate removal).

Five new measurements of average annual ipsilateral stroke rate from five apparently eligible studies were identified, see Table 1 (103–107). Two of these included a major update of studies included in the 2009 meta-analysis (105, 107). Only the most recent data were used in this meta-analysis update (105, 107). Two others were identified which were otherwise eligible except that they included a minority of patients (≤18%) with remote ipsilateral stroke or TIA (>18–24 months prior to recruitment) (17, 108). Analyses were made with and without including these two studies. As before, temporal changes in ipsilateral stroke rate were sought using ordinary least squares linear regression analysis, weighted according to sample sizes, using the new and previously identified studies (3).

**Table 1:** Average Annual Ipsilateral “Stroke” Rates in studies considered for the 2013 updated meta-analysis of patients with moderate–severe (≥50%) asymptomatic carotid stenosis given medical treatment alone.^a,b^

Reference	Sample size	% Stenosis	Follow-up (years)	Ipsilateral stroke rate	Included in Figure 1?
Gronholdt et al. (103)	111	≥50 by US	4.4 mean	3.1	Yes
Marquardt et al. (104)	101	≥50 by US or MRI	3 mean	0.3	Yes
Markus et al. (17)	477	≥70 by US	2 for all patients	0.6	Yes^c^
Nicolaides et al. (105)	923	70–99% by US	4 mean	1.5	Yes
Silvestrini et al. (106)	162	60–99 by US	2.8 mean	2.9	No
Madani et al. (108)	253	≥60 by US	3 for all patients	0.7	Yes^c^
den Hartog et al. (107)	293	50–99 by US	6.2 mean	0.3	Yes

*^a^All ipsilateral stroke rates were calculated using raw data and only the first ipsilateral stroke/patient. Further, patients with a first ipsilateral stroke and a prior ipsilateral transient ischemic attack (TIA) during follow-up were excluded when this distinction was possible. US, ultrasound; MRI, magnetic resonance imaging*.

*^b^Definitions of stroke and TIA were clinically based except in the study by Silvestrini et al. which was imaging-dominant (106). Definitions of stroke in all other studies also included a 24-h differentiation from TIA (personal communication from Doctors Hugh Markus and Gert deBorst), except in the earliest study by Johnson et al. in which the time distinction between TIA and stroke was not defined (109)*.

**Figure 1:**
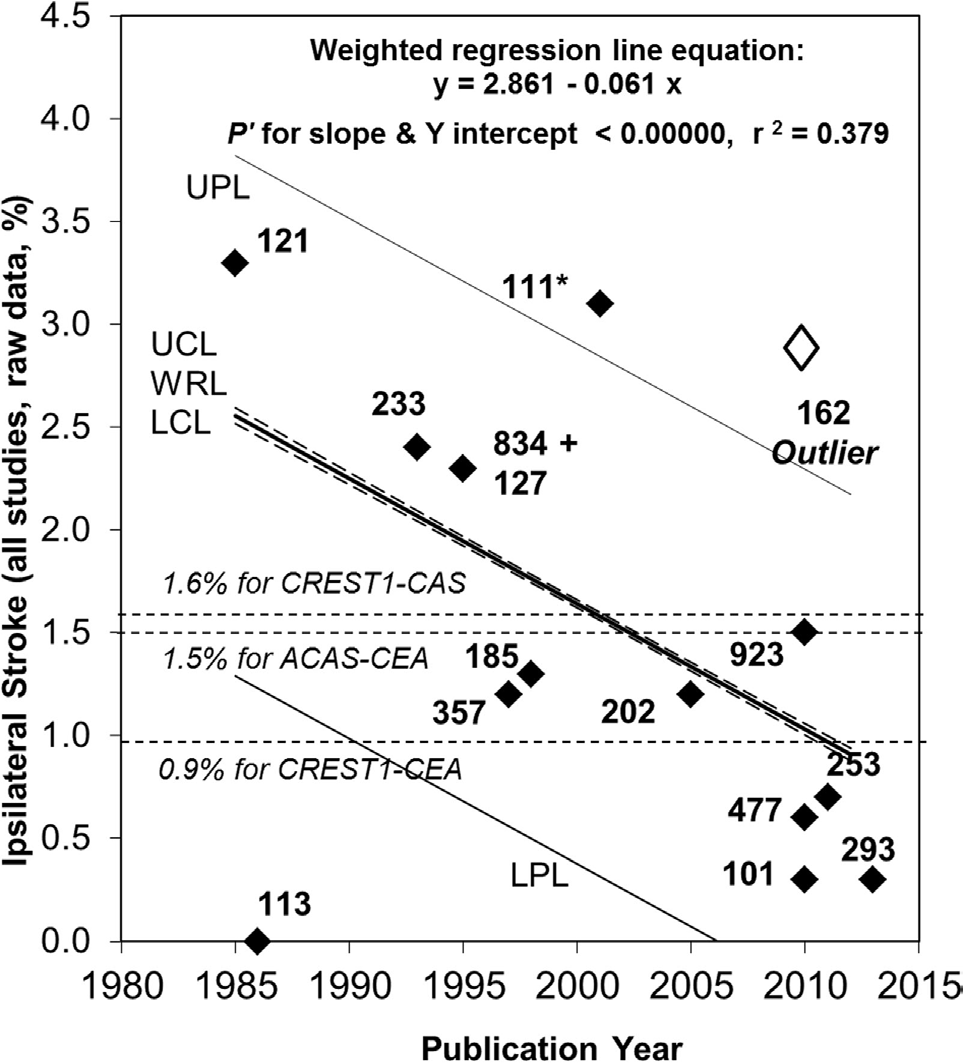
Continued fall in the rate of ipsilateral stroke in patients with >50% carotid stenosis given medical treatment alone since 2007. Sixty-seven percent relative (or 1.7% absolute) fall in the reported average annual ipsilateral stroke rate in patients with >50% ACS given medical intervention alone from 1985 to 2013. Black diamonds are study results with corresponding sample sizes. As in 2009, the Ryan-Holm stepdown Bonferroni correction was made for multiple comparisons (converting raw *P* values to *P*′ values, SYSTAT 13, SYSTAT Software Inc.) (3). UPL and LPL, respectively, upper and lower 95% prediction limits for new population rate estimates; UCL and LCL (dashed lines), respectively = upper and lower 95% confidence limits for the population regression line; WRL, weighted regression line; ACAS, Asymptomatic Carotid Atherosclerosis Study (112); CREST1, Carotid Revascularization Endarterectomy versus Stenting Trial (113). ** indicates two studies including a small minority with remote ipsilateral stroke/transient ischemic attack at baseline (17, 108). These were included in this analysis because the regression line was not very different with (y = 2.861 – 0.061x, *P*′ for slope and y intercept <0.00000 and r^2^ = 0.379) or without them (y = 2.720 – 0.051 x, *P*′ for slope and y intercept <0.00000 and r^2^ = 0.275). Both analyses showed a statistically significant fall in stroke risk from 1985 to 2013 although it was a little less in magnitude when the studies by Markus et al. (17) and Madani et al. (108) were excluded (57% relative and 1.4% absolute fall in rate). The outlier result (white diamond) (106) was not included in these analyses (see text). Abbreviations: CEA, carotid endarterectomy; CAS, carotid stenting.

*^c^Did not meet the meta-analysis inclusion criteria for minor reasons (see text and Figure 1). Analyses were performed with and without the results of these two studies*.

With respect to change in the average annual ipsilateral stroke rate over time, there was a notable outlier with a statistically significant, approximately five times higher stroke rate compared to most other studies also published around 2010, see Figure 1 (106). The reason was the use of proposed new stroke and TIA definitions from Easton et al. (19) (personal communication with Mauro Silvestrini). As mentioned, these proposed definitions were ambiguous. However, in the study by Silvestrini et al., it meant that stroke was defined as “focal events (including transient) in which neuroimaging [MRI or CT] identified an ischemic lesion.” No “imaging-negative” deficits were noted. Therefore, the “stroke” definition used in that study (and the follow-up paper) (110) was consistent with what others had called “stroke or TIA.” The results from Silvestrini et al. had to be removed from this analysis to avoid artificial over-estimation of stroke risk using medical treatment alone. Meanwhile, all other studies in Figure 1 used a clinically based definition of stroke. Two other outlying results from relatively small studies were attributed to random variation (103, 111).

Attempts to use the AHA/ASA proposals highlight the vital importance of always providing complete definitions of stroke and TIA, particularly definitions that are unambiguous and universally applicable (at least at a fundamental level). Moreover, attempts to use the AHA/ASA proposals force incompatibility with all past measurements of stroke and TIA rate (101). In this instance, the documentation of a continuing fall in the risk of ipsilateral stroke in patients with asymptomatic carotid stenosis given medical treatment alone (lifestyle coaching and medication) was almost lost because of attempts to use the AHA/ASA proposals. This kind of significant problem is avoidable if we continue to record the persistence, or not, of clinical deficits beyond 24 h (a practical time-point) (20, 100) and sub-classify patients according to this timing and, where appropriate, then according to the neuroimaging findings.

## The Proposals are Otherwise Clinically Unhelpful

### Limitations Imposed by Access to Neuroimaging

The proposed definitions are constrained by access to acute state-of-the-art brain imaging, specialized acute stroke services and patient compatibility with certain imaging techniques. Stroke and imaging services are most available in high-income countries and, even there, access is variable (99, 114). Further, approximately 70% of strokes world-wide occur in low-middle income countries (115). Hence, the proposed imaging-dominant definitions would be applicable to a very biased selection of patients. To define all stroke and TIA by a subcategory of imaged patients is inappropriate. By contrast, the established clinically based definitions are accessible to all patients because they are dependent only on clinical status with sub-classification as resources allow.

### Negative Patient Psycho-Social Implications

The negative psycho-social implications of the proposals need to be considered. With a growing focus on imaging alone to diagnose stroke, we are seeing more patients in clinical practice with non-specific symptoms and non-specific imaging findings being diagnosed with stroke when this is not justified. Further, patients who would have previously been diagnosed with TIA are now being diagnosed with stroke because of imaging findings. Stroke, in contrast to TIA, implies the likelihood of persisting deficits. Persons diagnosed with stroke are expected to inform other clinicians, licensing authorities, insurance companies, potential employers, and others of their diagnosis. They are likely to be unfairly restricted if the diagnosis is inaccurate relative to the established clinically based definitions upon which the evidence base is derived. Such over-diagnosis of stroke is likely to cause avoidable anxiety (116) and exposure to unnecessary stroke prevention treatments which carry risk and financial cost without benefit.

### Encouragement of Over- and Under-Treatment

Over-treatment is likely to be compounded by the AHA/ASA proposed lumping together of asymptomatic and symptomatic patients with brain imaging findings under a diagnosis of stroke. As already discussed, imaging can only ever provide evidence of infarction. MRI evidence of brain lesions, commonly referred to as “infarction,” in asymptomatic people becomes increasingly common with age and is also highly dependent on reporting methods (36, 117). Further, the asymptomatic and symptomatic states should be distinguished as clearly as possible because they differ so much with respect to prognosis and potential gain from intervention (118). Under-treatment is also encouraged by the proposals. “Imaging-negative” TIA or stroke patients (without “positive” neuroimaging evidence of ischemia) may not be recognized as likely to benefit from treatment. They risk being denied proven therapies according to a large evidence base established using clinically based definitions and that was not reliant on detecting “positive” neuroimaging evidence of ischemia (5, 6, 51, 119).

## The Proposals Do not Solve the Challenges of Delivering Hyperacute Stroke Therapy

### Reasons Given to Change to Imaging-Dominant Definitions

Reasons given for the AHA/ASA proposals relate to the challenges of administering hyperacute ischemic stroke therapy and are listed below:

- The 24-h symptom duration misclassifies up to one-third of patients who have experienced tissue infarction as not having tissue infarction (19).
- TIA is not seen as urgent (18).
- A 24-h limit for transient cerebral ischemia is arbitrary and not reflective of the typical TIA duration (usually <30–60 mins) (18).
- A 24-h differentiation has the potential to delay effective hyperacute stroke therapies (18, 19).
- There is no biological justification to continue to treat the 24-h time-point as particularly helpful to recognize (19).
- Physicians need to focus on the cause of ischemia rather than duration of symptoms (18, 19).
- A “tissue-based” definition of TIA encourages use of neurodiagnostic tests (19).

### How These Issues Should Be Addressed

These perceived challenges will not be addressed by changing to imaging-dominant definitions of stroke and TIA. For the reasons already given, neuroimaging is unreliable for identifying histological infarction or for providing a “tissue-based” diagnosis. Neuroimaging provides a radiologically based diagnosis (or, more accurately, a radiologically based part of the diagnosis). Rather, the best approach is similar to non-invasively diagnosing acute myocardial infarction (also a pathologically defined tissue state). Here, clinical features, electrocardiographic changes, and cardiac enzyme levels, which are limited individually, have additive diagnostic value (120).

In particular, most patients (about 65%) with clinical features of acute focal brain ischemia lasting <24 h have no corresponding DWI or other imaging evidence of ischemia (99). DWI findings in patients with acute clinical features of brain ischemia are more likely with increasing deficit duration and severity and other clinical and pathophysiological markers of poorer outcome (55, 78, 121, 122). We acknowledge reports that DWI-positive patients (including particularly those with clinical deficits lasting less than 24 h) have a poorer outcome than DWI-negative patients (55, 78, 121, 122). However, imaging results must be shown to independently predict outcome, despite all other predictive clinical and pathophysiological markers. Moreover, even if ‘positive’ imaging evidence of ischaemia (or the nature of it) (123) proves to be such an independent predictor, treatment decisions are not currently altered (51). Even if patient outcomes are somehow shown to improve by using advanced imaging techniques and detecting “positive” evidence of ischemia, cost-effectiveness must be measured (119). Further, changes in definition of stroke and TIA will not necessarily be required to exploit such knowledge. Moreover, “imaging-positive” patients are likely to remain a subset of patients with acute central-neurovascular syndromes. Therefore, it is important to maintain the clinical focus and subcategorize according to available neuroimaging results.

There is no reason why the established clinically based definitions of stroke and TIA should cause complacency or delay appropriate therapy. Such problems are best addressed by education. The public and clinicians should be educated that to optimize patient outcomes, hyperacute therapies (such as thrombolysis and/or clot evacuation) should be administered as soon as possible within the first 4–6 h of clinical onset (5, 6). This is because imaging evidence of brain damage and the likelihood of a permanent deficit is seen in proportion to the duration of the deficit. Further, it should be acknowledged that in trials of thrombolysis and clot evacuation it was likely that some patients would have improved spontaneously and this was not predictable. However, overall, patients were better with treatment. It needs to be communicated that the aim in trials and clinical practice is to improve overall chances of converting long-term deficits (strokes) into transient ones (TIAs) and severe deficits into milder ones. Therefore, delaying transfer to hospital or hyperacute therapy to see if a deficit resolves is not justified. Further, the established definitions do not need to hinder pathophysiological work up.

Finally, at least one universally standardized time-point is required to differentiate short-lived, fully resolved clinical deficits from long-term ones, whether or not there has been any infarction or hyperacute therapy. As already explained, there is great clinical value in continuing this. There is no compelling data showing that an alternative time threshold is superior to, or more useful than 24 h (20). Further, the shorter the time-point used to differentiate stroke (long-term deficits) from TIA (short-term deficits) the harder it is to administer treatment to abort “stroke.” Anything less than 24 h will still be subject to the problems of being sure about how much tissue infarction has occurred. Moreover, the deficit duration and severity are more important than proving the presence or not of infarction and should remain the primary targets of treatment strategies. The 24-h time-point should be retained because it predominates in past research and is practical and useful to measure, whether or not patients are admitted to hospital or they receive hyperacute therapy. Additional times may be documented where there is reason.

## Conclusion and the Way Forward

### The Importance of Clinically Based Definitions

In conclusion, the patient’s clinical status (nature and duration of deficits) needs to remain the basis of stroke and TIA definitions because this is what matters most and has been the main outcome of prognostic and therapeutic studies. Further, definitions focused on the clinical deficit allow inclusion of all affected persons, reduce the risk of mis-diagnosis, and encourage continued collection of data regarding asymptomatic as distinct from symptomatic patients, and typical stroke and TIA patients as distinct from other presentations which may have different causation. Simplified clinical assessment and imaging interpretation cannot replace assessment by a stroke specialist (119). The value of a highly experienced expert physician is priceless and should be more highly valued by the profession as well as by patients.

The duration of the clinical deficit with respect to lasting more or less than 24 h should still always be recorded so that brief resolving deficits can be systematically distinguished from lasting ones, whether or not hyperacute stroke therapies are administered. TIA and stroke, respectively, define outcomes up to and beyond 24 h of clinical onset. Therefore, they are used retrospectively. Terms like “acute central-neurovascular syndrome” (19) or “threatening stroke” may help with communication during the first 24 h of clinical onset. However, patients fundamentally want to know if they have a stroke-related deficit and the likelihood that treatment may help resolve or minimize it.

Brain imaging remains a critical tool to characterize and sub-classify clinically defined stroke and TIA patients and help determine appropriate therapy. It is a significant advance that modern neuroimaging is now more likely to provide positive evidence of neural ischemia or infarction rather than to only help exclude other pathological processes, such as bleeding. However, the limitations of neuroimaging (especially if used as the predominant or only source of information) must be kept in mind. Further, infarction should remain a pathological term, allowing recognition that it is not reliably identified by neuroimaging and that accurate identification of infarction is not usually required to optimize patient outcomes. Challenges regarding the new era of hyperacute stroke therapy are best faced through appropriate education and resource organization. The AHA/ASA proposals to merge the established clinically based definitions of stroke and TIA with each other and with pathologically defined infarction according to “positive” imaging findings must not be accepted. The AHA/ASA proposed new “definitions” are ambiguous, scientifically incorrect, research disrupting, otherwise clinically unhelpful, and do not solve the problems they were proposed to solve.

### Improving the Use of Clinically Based Definitions

Research, and its application in clinical practice, requires standard definitions, and these need to be scientifically valid and optimized with respect to clinical relevance. Although the established clinically based definitions of stroke and TIA provide the best foundation (using sub-classification according to the available results from imaging and/or other investigations), use of these terms should be improved by:

- providing detail about typical clinical presentations and if/how stroke mimics were distinguished (34, 119).
- specifying the inclusion, or not, of stroke and TIA of the retina, brain and/or spinal cord and
- specifying as clearly as possible the inclusion, or not, of syndromes due to arterial and/or venous infarction or hemorrhage (20).

**Figure 2:**
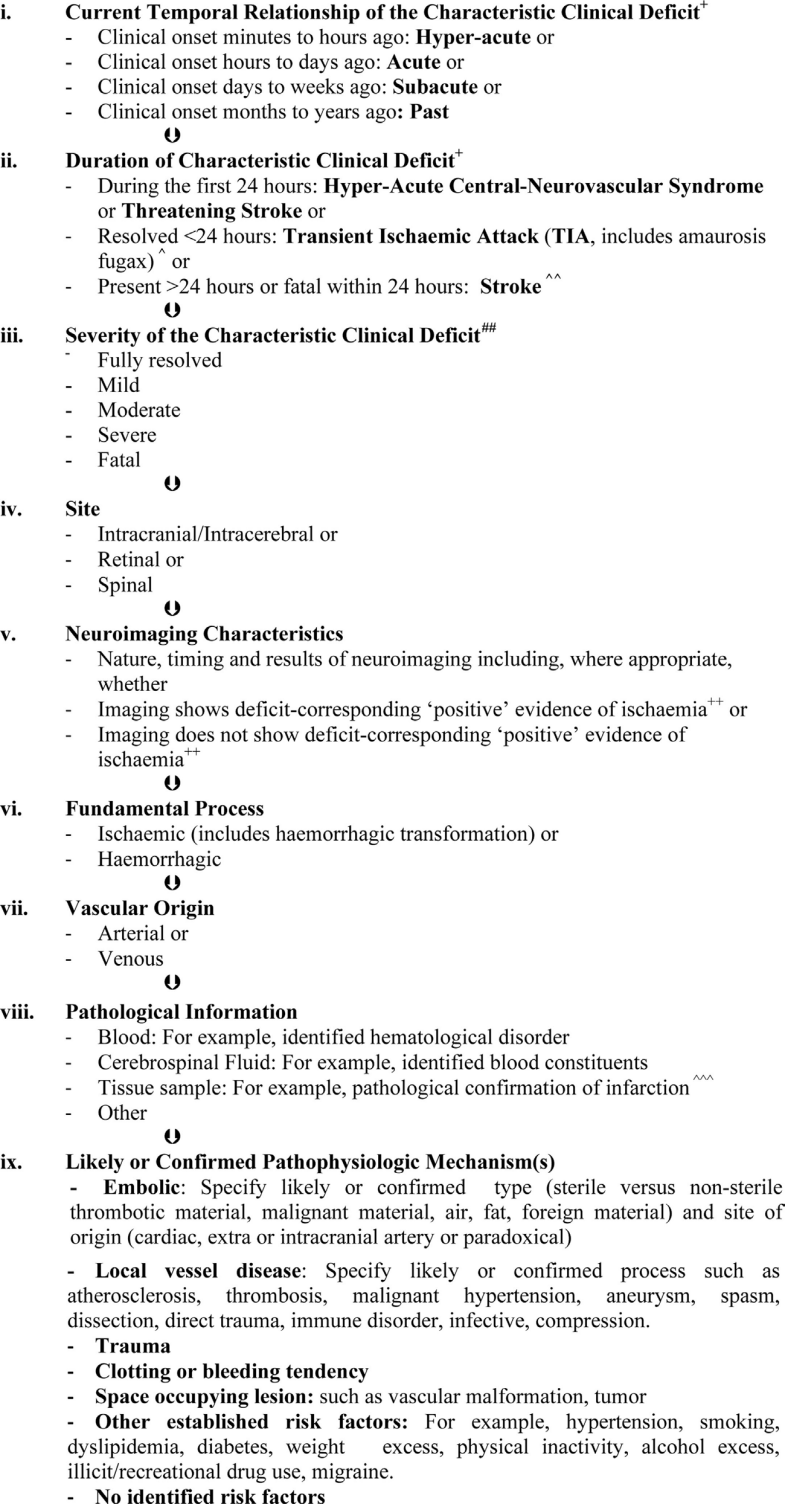
Diagnostic and classification framework for syndromes due to focal, intrinsic, central-neurovascular compromise*^,^**^,#^. *These are the sequential layers of information that should be specified as far as possible when describing syndromes due to focal, intrinsic central-neurovascular compromise. These are syndromes referable to intrinsic disorders of particular blood vessels supporting the brain, retina, or spinal cord. This classification may be expanded when there is reason. As far as possible, qualifying terms such as “likely” or “confirmed” should be included to improve clarity. **This framework recognizes that the presence of a condition does not always mean causation (124). The mechanism of stroke or transient ischemic attack (TIA) in an individual is always about assigning probabilities (0–100%). Therefore, it is more accurate to speak of risk factors and their likeliness of contributing causation. ^#^This is a classification of clinical syndromes (symptoms and/or signs). Asymptomatic neuroimaging findings should be classified separately and according to whether or not pathological characterization has occurred. Pathologically unconfirmed asymptomatic neuroimaging findings, such as suspected asymptomatic infarcts, should be classified as such with the reasons for suspecting a particular pathology. Asymptomatic neuroimaging findings can be sub-classified using the same principles as presented in Figure 2 where relevant, when known and when there is purpose. ^+^The most commonly recognized “characteristic” “focal” clinical deficits associated with stroke and TIA include sudden facial weakness, weakness or incoordination involving one or more limbs, dysphasia, dysarthria, visual impairment, vestibular or cranial nerve dysfunction which are manifested according to the particular vascular territory involved and neural tissue being secondarily compromised (7, 12, 13, 15). ^++^For computed tomography, these include hypo-attenuation relative to “normal” areas, loss of white/gray matter differentiation, focal cortical swelling, hyper-dense arterial signs, and/or compression of adjacent structures (25, 26). For magnetic resonance imaging these include diffusion-weighted, T2, and fluid attenuation inversion recovery hyperintensity (26, 39, 46). ^^^TIA is rapidly developed clinical symptoms and/or signs of cerebral, retinal, or spinal cord dysfunction lasting <24 h, with no apparent cause other than of focal neurovascular origin where resolution is swift and leaves no detectable permanent neurologic deficit. It is recognized that transient ischemic attacks commonly last 2–15 min. Adapted from the National Institutes of Health 1975 publication (12). ^^^^Stroke is rapidly developed clinical symptoms and/or signs of cerebral, retinal, or spinal cord dysfunction lasting >24 h or leading to death, with no apparent cause other than of focal neurovascular origin. It is recognized that the deficits typically appear suddenly but may progress or fluctuate (not resolving), particularly over minutes to hours after onset. Adapted from Aho et al. (7). ^^^^^Infarction is a pathological term used to describe tissue that has lost its blood supply for long enough to undergo ischemic necrosis (death) with characteristic macroscopic and microscopic findings (21). ^##^Severity may be measured according to meaningful scales such as the modified Rankin (for activities of daily living) (125). “Fully resolved, mild, moderate, severe, or fatal” should be used in preference to “disabling” versus “non-disabling” to describe severity because even mild strokes are associated with disability unless all measurable deficits have resolved.

In addition, it should not be forgotten that episodes of apparent global dysfunction (such as impaired consciousness) may occasionally be caused by ischemic or haemorrhagic compromise directly referable to one or more cerebral artery (8–11). A summary of these and other proposed improvements for using the established clinically based definitions of stroke and TIA is given in Figure 2. This framework is universally accessible at the most fundamental levels and, at the same time, can be used as we advance further into “personalized” (more individually tailored) medicine.

## Author Note

Anne L. Abbott, Mauro Silvestrini, Raffi Topakian, Jonathan Golledge, Alejandro M. Brunser, Gert J. de Borst, Robert E. Harbaugh, Fergus N. Doubal, Tatjana Rundek, Ankur Thapar, Alun H. Davies, Anthony Kam, and Joanna M. Wardlaw are members of the Faculty Advocating Collaborative and Thoughtful Carotid Artery Treatments (FACTCATS at FACTCATS.org).

## Author Contributions

All authors contributed to the writing, editing, and final approval of this manuscript.

## Conflict of Interest Statement

AA is a neurologist and receives a part-time salary from the Bupa Health Foundation to continue independent activities to improve outcomes for patients at risk of stroke and other complications of arterial disease. RT serves on the advisory board for Novartis and Shire-Baxalta. He has received support for Conference attendance from Novartis, Pfizer, Abbvie, and Bayer and Speaking honoraria from Pfizer. JG is supported by an NHMRC Practitioner Fellowship and funding from the NHMRC and Queensland Government. GB is a member of the steering committee ECST-2 trial. FD receives research salary funding from the Stroke Association and Garfield Weston Foundation. TR holds National Institutes of Health (NIH) grants. AD holds a number of National Institute for Health Research grants. JW has no current grants that are of direct relevance to this paper. However, she holds other grants for research in stroke and aging and dementia that are managed by the University including from the MRC, Engineering and Physical Sciences Research Council, Wellcome Trust, Fondation Leducq, European Union Horizon 2020 fund, Stroke Association, British Heart Foundation and Alzheimer’s Society. All other authors declare that the research was conducted in the absence of any commercial or financial relationships that could be construed as a potential conflict of interest.
